# Cryptolepine inhibits melanoma cell growth through coordinated changes in mitochondrial biogenesis, dynamics and metabolic tumor suppressor AMPKα1/2-LKB1

**DOI:** 10.1038/s41598-017-01659-7

**Published:** 2017-05-04

**Authors:** Harish C. Pal, Ram Prasad, Santosh K. Katiyar

**Affiliations:** 1 Department of Dermatology, University of Alabama at Birmingham, Birmingham, AL USA; 2 Environmental Health Sciences, University of Alabama at Birmingham, Birmingham, AL USA; 3 Comprehensive Cancer Center, University of Alabama at Birmingham, Birmingham, AL USA; 4 Birmingham Veterans Affairs Medical Center, Birmingham, AL USA

## Abstract

Dysregulated mitochondrial dynamics and biogenesis have been associated with various pathological conditions including cancers. Here, we assessed the therapeutic effect of cryptolepine, a pharmacologically active alkaloid derived from the roots of *Cryptolepis sanguinolenta*, on melanoma cell growth. Treatment of human melanoma cell lines (A375, Hs294t, SK-Mel28 and SK-Mel119) with cryptolepine (1.0, 2.5, 5.0 and 7.5 μM) for 24 and 48 h significantly (*P* < 0.001) inhibited the growth of melanoma cells but not normal melanocytes. The inhibitory effect of cryptolepine was associated with loss of mitochondrial membrane potential and reduced protein expression of Mfn1, Mfn2, Opa1 and p-Drp1 leading to disruption of mitochondrial dynamics. A decrease in the levels of ATP and mitochondrial mass were associated with activation of the metabolic tumor suppressor AMPKα1/2-LKB1, and a reduction in mTOR signaling. Decreased expression of SDH-A and COX-I demonstrated that cryptolepine treatment reduced mitochondrial biogenesis. *In vivo* treatment of A375 xenograft-bearing nude mice with cryptolepine (10 mg/Kg body weight, *i.p*.) resulted in significant inhibition of tumor growth, which was associated with disruption of mitochondrial dynamics and a reduction in mitochondrial biogenesis. Our study suggests that low toxicity phytochemicals like cryptolepine may be tested for the treatment of melanoma.

## Introduction

The mitochondrion is the most important cellular organelle involved in the processes of bioenergetics and biosynthesis and plays a key role in cellular signaling. Vital cellular functions such as regulation of energy production, maintenance of redox status and cell growth are controlled by mitochondria^1–3^. Shifts in mitochondrial function may lead to impaired biosynthetic pathways, dysregulated cellular signal transduction pathways, and modulation of gene transcription as well as chromatin structure^4, 5^. Alterations in mitochondrial mass or functions influence cellular functions, proliferation, and survival, and have been shown to affect metastasis as well as the clinical outcome of patients with cancer^2, 6^. Mitochondrial biogenesis in cancer is influenced by the tumor type, tumor microenvironment and the metabolic state^2, 7^. The process of mitochondrial biogenesis is controlled by various genes expressed in both the nuclear genome and the mitochondrial genome^2, 8^. It has been suggested that the transcription factor c-Myc that regulates cell proliferation, cell cycle, metabolism and apoptosis is a central regulator of mitochondrial biogenesis as loss of c-Myc reduces mitochondrial biogenesis and, conversely, gain of c-Myc increases mitochondrial biogenesis^9^. Expression of Sirtuins (SIRT1 and SIRT3) also is related to enhanced tumorigenesis and metastasis through their regulation of mitochondrial biogenesis and activation of peroxisome proliferator-activated receptor gamma coactivator 1-alpha (PGC-1α)^10, 11^. AMP-activated protein kinase (AMPK), which is a metabolic tumor suppressor, acts as an energy sensor^12, 13^ and maintains energy homeostasis as well as regulating various processes associated with tumor development such as cell growth, cell survival, cell cycle progression and apoptosis^13, 14^.

Cutaneous melanoma is the most lethal form of skin cancer. The American Cancer Society estimated that in the United States in the year 2016 approximately 76,380 new cases of cutaneous melanoma were diagnosed and approximately 10,130 individuals were died of this disease^15^. Melanoma develops from neoplastic melanocytes which are constantly exposed to sunlight specifically in fair skin individuals. A growing body of evidence indicates that increased mitochondrial biogenesis in melanoma cells results in enhanced tumorigenesis and metastasis as well as drug resistance to inhibitors of the mitogen-activated protein kinases (MAPK)^3, 16, 17^. Thus, inhibition of mitochondrial biogenesis can be considered as a potentially novel approach to inhibition of melanoma development and progression.

Cryptolepine, a pharmacologically active plant alkaloid, is isolated from the roots of the shrub *Cryptolepis sanguinolenta* (Lindl.) commonly found in the Central and Western regions of the African continent^18^. In various biochemical and pharmacological assays, it has been demonstrated that cryptolepine has significant potential as an anti-malarial, anti-bacterial, and anti-hyperglycemic agent under different *in vitro* and *in vivo* conditions^19–21^. Some studies have reported a significant anti-inflammatory potential. Both inflammatory mediators, such as COX-2/PGE_2_ signaling, and promoters of inflammation, including TNFα and iNOS, have been identified as potential targets of the anti-inflammatory action of cryptolepine^22–24^. The anticancer effects of cryptolepine have been reported as due to its direct interactions with DNA, inhibition of DNA synthesis and inhibition of topoisomerase functions^25–27^. However, the molecular mechanisms underlying the potential cytotoxicity against cancer cells and in particular melanoma are not known and have not been explored. Therefore, we investigated the anti-cancer potential of cryptolepine using human melanoma cells. We report that treatment of human melanoma cells with cryptolepine inhibits the growth and viability of melanoma cells in culture and in an *in vivo* mouse xenograft model and does so by targeting the mechanisms that regulate mitochondrial dynamics and mitochondrial biogenesis.

## Results

### Cryptolepine reduces the viability of melanoma cells but has less effect on normal human melanocytes

**Figure 1:**
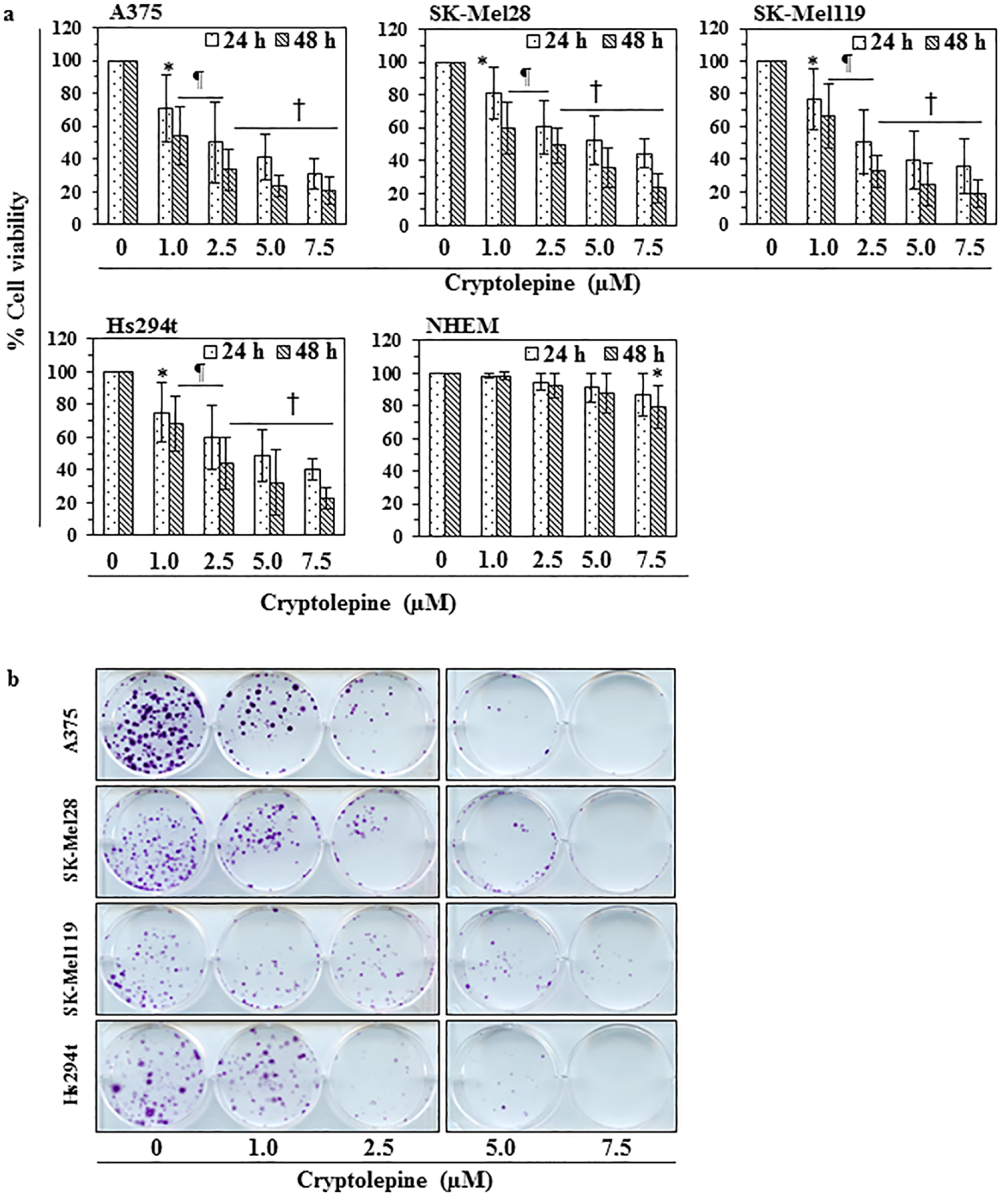
Cryptolepine treatment inhibits the viability and growth of melanoma cells. (**a**) Treatment of melanoma cells (A375, SK-Mel28, SK-Mel119 and Hs294t) and normal human epidermal melanocytes with cryptolepine (0, 1.0, 2.5, 5.0 and 7.5 µM) for 24 or 48 h inhibits cell viability in a dose- and time-dependent manner. Cell viability was determined using an MTT assay as described in the Materials and Methods. Cells were seeded in 96-well plates in 6 replicates/sample and per treatment group. Cells treated with DMSO alone served as a vehicle control. Statistical significance versus control group, *P < 0.05, ^¶^ *P* < 0.01, ^†^ *P* < 0.001. **(b)** Effect of cryptolepine on the colony forming capacity of melanoma cells. Colonies were detected by staining with crystal violet and plates were scanned for photographs. Colonies appear blue-violet.

We first determined the short-term effects of cryptolepine on the viability of various human melanoma cell lines (*i.e*., A375, Hs294t, SK-Mel 28 and SK-Mel 119) using different concentrations of cryptolepine (0, 1.0, 2.5, 5.0 and 7.5 µM) for 24 and 48 h. An MTT assay revealed a dose-dependent decrease in the viability of A375 cells after treatment with cryptolepine for 24 h (13.7 to 40.8%; *P* < 0.05–*P* < 0.01) and after 48 h (15.7 to 52.8%; *P* < 0.05–*P* < 0.001) (Fig. 1a). Under identical experimental conditions, similar effects of cryptolepine were observed on treatment of Hs294t, SK-Mel28 and SK-Mel119 cells. The sensitivity of normal human epidermal melanocytes (NHEM) to the cytotoxic effects of cryptolepine was significantly lower than the sensitivity of the melanoma cell lines (*P* < 0.01–*P* < 0.001) (Fig. 1a).

### Cryptolepine reduces the clonogenic potential of melanoma cells

To determine the long-term cytotoxic effect of cryptolepine treatment on melanoma cells, a colony formation assay was employed. For this purpose, melanoma cells (A375, Hs294t, SK-Mel28 and SK-Mel119) were treated with cryptolepine. As shown in Fig. 1b, cryptolepine significantly inhibited colony formation by the melanoma cell lines. The reduction in colony formation in terms of both size and numbers was dependent on the concentration of cryptolepine used to treat the cells prior to the colony formation assay. These data provide further evidence that cryptolepine has a cytotoxic effect on human melanoma cells.

### Cryptolepine induces loss of mitochondrial membrane potential in melanoma cells

**Figure 2:**
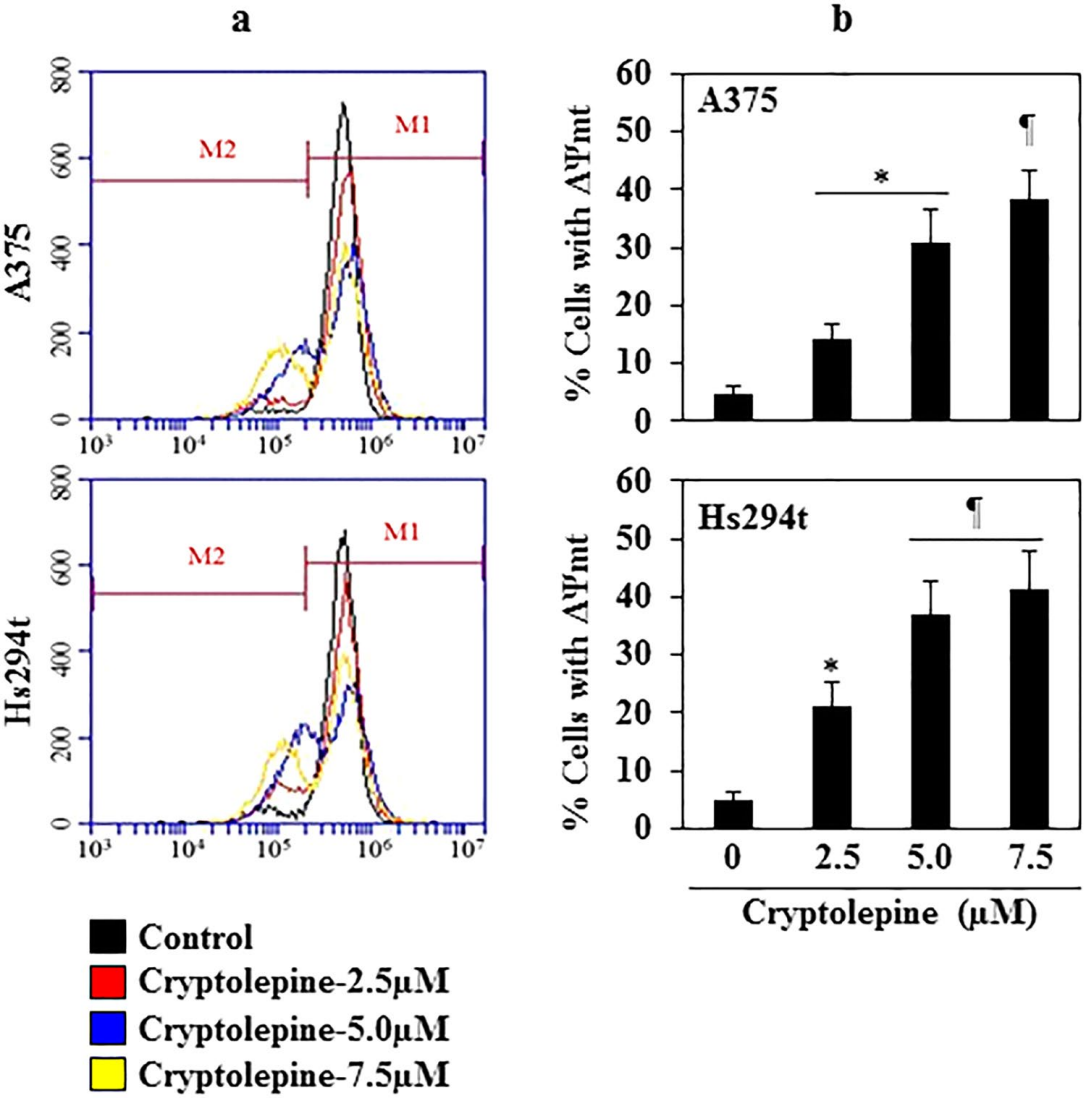
Treatment with cryptolepine disrupts mitochondrial membrane potential in melanoma cells. (**a**) Representative melanoma cell lines (A375 and Hs294t) were treated with various concentrations of cryptolepine (0, 2.5, 5.0 and 7.5 µM) for 24 h. Thereafter, cells were harvested, incubated with Rhodamine 123 and then analyzed for loss of mitochondrial disruption using flow cytometry. Data were compared with non-cryptolepine-treated cells. Treatment groups are shown in color. **(b)** Percentage of cells with mitochondrial disruption in different treatment groups was determined. The resultant data are presented in terms of the percentage of cells with loss of mitochondrial membrane potential and as means ± SD, n = 3. Statistical difference versus control, *P < 0.05 and ^¶^ *P* < 0.01.

To determine whether the cryptolepine-induced reduction in melanoma cell viability or toxicity was associated with disruption of mitochondrial integrity and function, we determined the effect of cryptolepine on mitochondrial membrane potential using the Rhodamine 123 fluorescent probe. As the cytotoxic effect of cryptolepine on the viability of different melanoma cell lines was similar, we selected two melanoma cell lines (A375 and Hs294t) for further experiments. The melanoma cells were treated with various concentrations of cryptolepine (0, 2.5, 5.0 and 7.5 µM) *in vitro* and the numbers of Rhodamine 123-stained cells quantified using flow cytometry. We found a significant decrease (*P* < 0.01) in Rhodamine-123-positive cells in the cryptolepine-treated A375 and Hs294t melanoma cells, suggesting that cryptolepine induces a loss of mitochondrial membrane potential in these cells (Fig. 2a). Depending on the dose of cryptolepine, the percentage of A375 cells with a reduced mitochondrial membrane potential was 14.1 to 38.4% (*P* < 0.05–*P* < 0.01) as compared to 4.8 percent of vehicle-treated control cells. In Hs294t cells, the percentage of cells with loss of mitochondrial membrane potential was 21.2 to 41.2% (*P* < 0.05–*P* < 0.01) as compared to 5.0% of vehicle-treated control cells (Fig. 2b).

### Cryptolepine reduces the levels of proteins involved in mitochondrial dynamics (fusion/fission)

**Figure 3:**
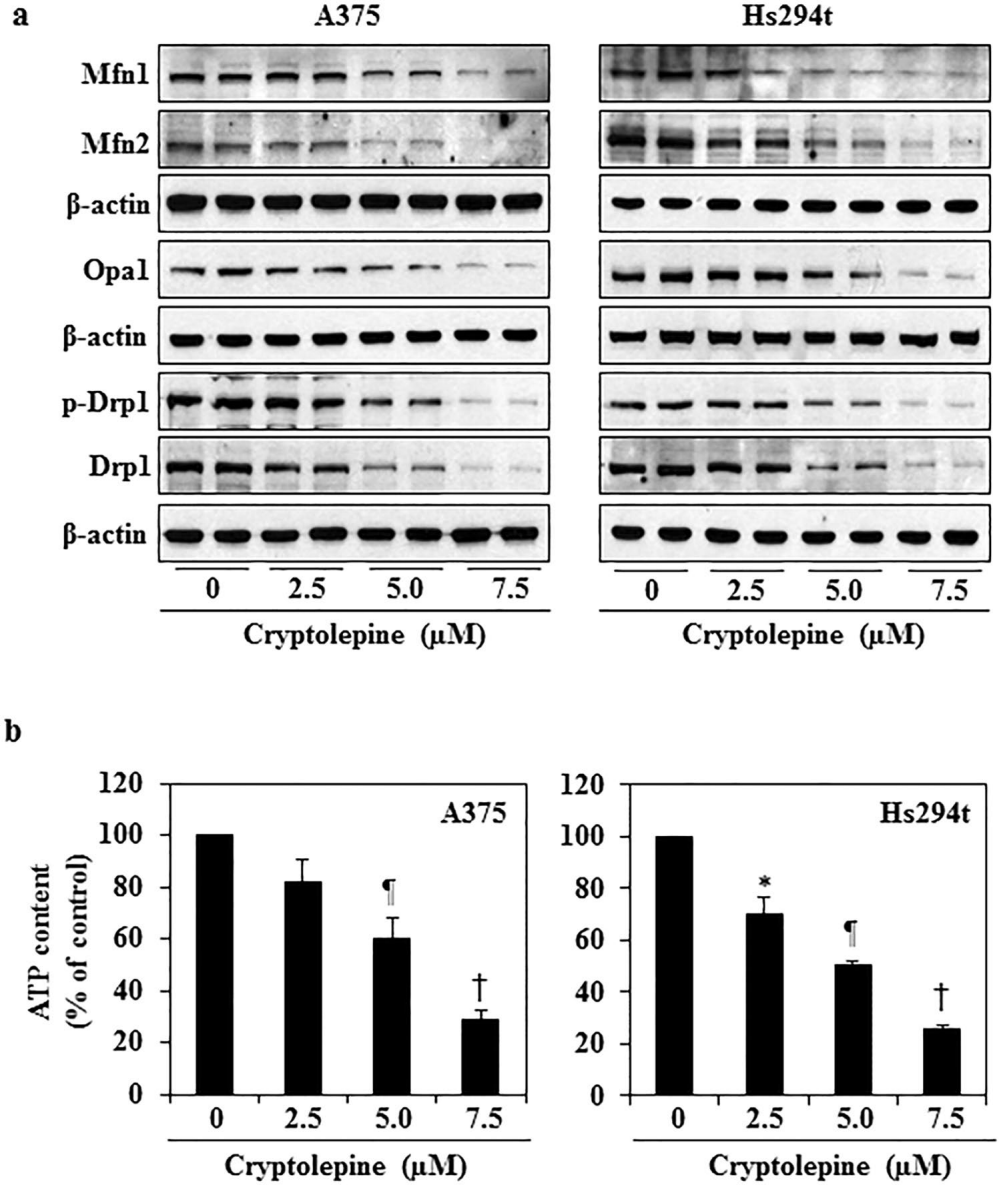
Treatment with cryptolepine reduces the protein levels of mitochondrial dynamics and decreases ATP levels. A375 and Hs294t melanoma cells were treated with cryptolepine (0, 2.5, 5.0 and 7.5 µM) for 24 h. (**a**) Cell lysates were prepared and subjected to analysis of mitochondrial fission and fusion proteins. Equal loading of proteins on the gels was verified by stripping the membrane and reprobing with β-actin. (**b**) ATP production was determined in cell lysates using an ATP determination kit, as described in Material and Methods. The experiment was performed in triplicate and results are expressed as mean ± SD in terms of percentage of control. Statistical significance versus control, *P < 0.05, ^¶^ *P* < 0.01 and ^†^ *P* < 0.001.

As treatment of cryptolepine induces loss of mitochondrial membrane potential in melanoma cells, we investigated whether this effect is due to its effects on mitochondrial fission or fusion proteins. It has been shown that the mitochondria shaping proteins regulate mitochondrial morphology and dynamics by modulating their size and their numbers^8, 28^. Mitofusins (Mfn1 and Mfn2) and the mitochondrial dynamin-like GTPase, Opa1, control mitochondrial dynamics through regulation of mitochondrial fusion whereas Dynamin-1-like protein (Drp1), which is also a GTPase, regulates mitochondrial fission^29, 30^. Western blot analysis revealed that treatment of A375 and Hs294t cells with cryptolepine (2.5, 5.0 and 7.5 µM) for 24 h resulted in a concentration-dependent decrease in the levels of Mfn1 and Mfn2 proteins as compared to vehicle-treated control cells (Fig. 3a). In addition, a dose-dependent decrease in Opa1 and Drp1 proteins was observed. It has been shown that overexpression of Drp1 is associated with cancer cell survival and tumor growth and inhibition of Drp1 in tumor cells has been associated with reduced cell viability and inhibition of tumor progression^28, 31^.

### Cryptolepine reduces the levels of cellular ATP in melanoma cells

To determine the cause of cryptolepine-induced mitochondrial membrane disruption, we tested the effects of cryptolepine on the levels of ATP in melanoma cells. The levels of ATP in melanoma cells were determined after treatment of the cells with various concentrations of cryptolepine (0, 2.5, 5.0 and 7.5 µM) for 24 h. We found that treatment of cryptolepine significantly decreased the levels of ATP in A375 cells (18 to 71%, *P* < 0.05 to *P* < 0.001) and in Hs294t cells (30 to 75%, *P* < 0.05 to *P* < 0.001) as compared to the levels of ATP in vehicle-treated control cells (Fig. 3b).

### Cryptolepine induces mitochondria depletion, activation of AMPKα1/2 and increases in LKB1 protein levels in melanoma cells

**Figure 4:**
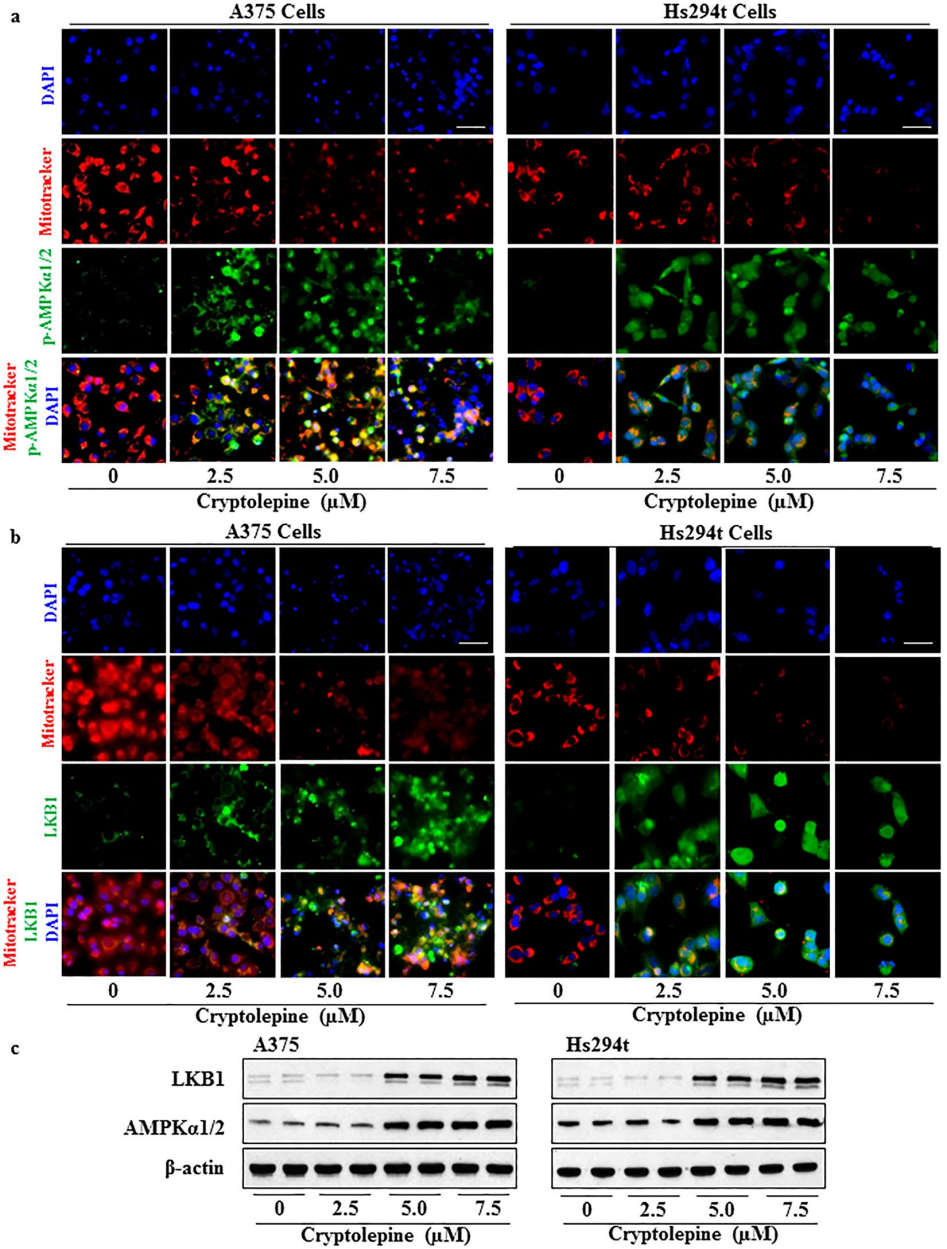
Cryptolepine treatment depletes mitochondria and increases the levels of AMPKα1/2 and LKB1 in melanoma cells. (**a** and **b**) Approximately 1x10^5^ A375 or Hs294t melanoma cells/well were plated in four-well chambered slides and treated with 0, 2.5, 5.0 and 7.5 µM cryptolepine for 24 h. Cells were stained with MitoTracker Red for 30 min. After fixation, permeabilization and blocking, cells were incubated with p-AMPKα1/2 or LKB1 antibodies overnight at 4 °C. Staining was performed as detailed in Materials and Methods. Images were acquired at 40x magnification using a Keyence Fluorescence Microscope BZ-X710 (Keyence Corporation of America). A representative photomicrograph from each group is shown. **(c)** After treatment of cells with cryptolepine for 24 h, total cell lysates were prepared and subjected to western blot analysis to determine the levels of AMPKα1/2 and LKB1 proteins. Blots were developed using chemiluminescence-specific ECL detection agents. Equal loading of proteins was verified by stripping the membrane and reprobing with anti-β-actin antibody.

**Figure 5:**
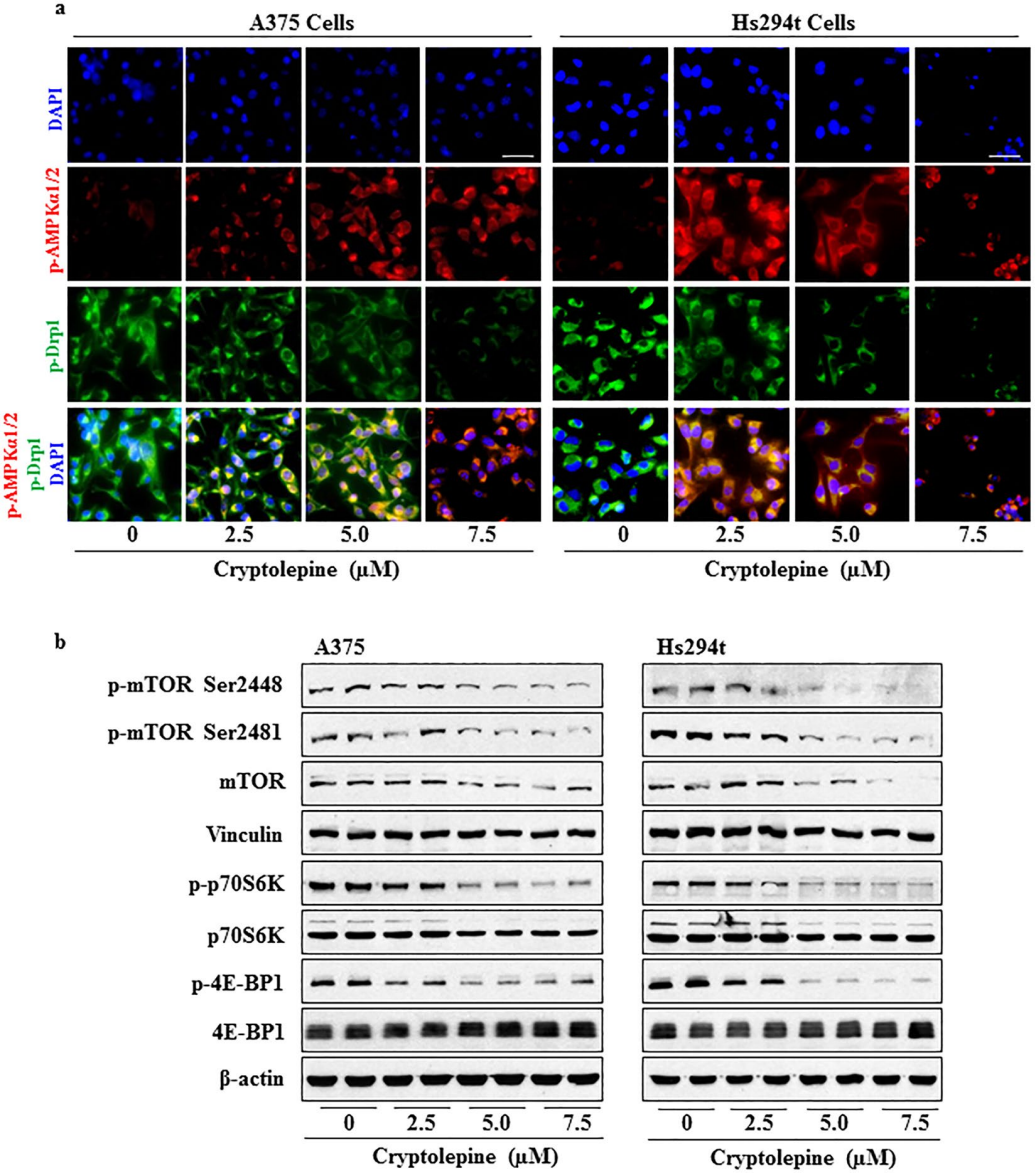
Treatment with cryptolepine disrupts mitochondrial dynamics by activating AMPK phosphorylation, reducing Drp1 phosphorylation and inhibiting mTOR signaling in melanoma cells. (**a**) Immunofluorescence staining of p-AMPKα1/2 and p-Drp1 was performed after the treatment of cells with cryptolepine, as detailed in Materials and Methods. Photomicrographs were obtained using Keyence Fluorescence Microscope BZ-X710 (Keyence Corporation of America). A representative photomicrograph from each group is shown. (**b**) After treatment of A375 and Hs294t cells with cryptolepine for 24 h, total cell lysates were prepared and subjected to western blot analysis to determine the level of proteins involved in mTOR signaling. Blots were developed using a chemiluminescence-specific ECL system. Equal loading of proteins was verified by stripping the membrane and reprobing with anti-β-actin or vinculin antibodies.

We then tested whether the cryptolepine-induced loss of mitochondrial membrane potential and ATP depletion affects the mass of mitochondria in melanoma cells and/or stimulates an increase in the levels of the energy sensing 5′ adenosine monophosphate-activated protein kinase (AMPKα1/2) and its upstream regulator, the liver kinase B1 (LKB1). Cryptolepine (2.5, 5.0 and 7.5 µM)-treated A375 and Hs294t melanoma cells were stained simultaneously with MitoTracker Red CMXRos dye and antibodies specific for p-AMPKα1/2 or LKB1. The MitoTracker Red CMXRos dye accumulates in healthy mitochondria in live cells. The number of red-fluorescent dye containing cells was markedly lower in cryptolepine-treated cells than the non-cryptolepine-treated control cells suggesting that cryptolepine treatment induces mitochondria depletion that contributes to the ATP depletion in A375 and Hs294t melanoma cells (Fig. 4a). Both the phosphorylation of AMPKα1/2 and the levels of LKB1 were increased in the A375 and Hs294t melanoma cells with damaged mitochondria in the cryptolepine-treated groups as compared to vehicle-treated control cells (Fig. 4a,b). Western blot analysis of the same treatment groups revealed that the treatment of melanoma cells with cryptolepine resulted in higher levels of AMPKα1/2 and LKB1 proteins as compared to vehicle-treated control cells (Fig. 4c). We also found that cryptolepine treatment reduced the levels of Drp1 protein as well as the levels of Drp1 phosphorylation with a concomitant increase in AMPKα1/2 phosphorylation in A375 and Hs294t melanoma cells in a concentration-dependent manner (Fig. 5a). These results provide strong evidence that cryptolepine reduces the viability of melanoma cells by targeting mitochondrial dynamics and inducing activation of AMPKα1/2 proteins.

### Cryptolepine inhibits protein synthesis by inhibiting mTOR signaling in melanoma cells

Protein synthesis in cells is considered to be one of the most energy consuming processes among those required for cellular growth and survival. The mechanistic target of rapamycin (mTOR) plays a crucial role in multiple cellular processes including protein synthesis, cell growth, cell cycle, cell survival, and autophagy^32^. mTOR has been shown to be activated in the majority of malignant melanomas^33, 34^. Cross-talk between AMPK and mTOR signaling is crucial for regulating cellular metabolism, energy homeostasis, cell growth and cell survival and a growing body of evidence indicates that AMPK activation negatively regulates the mTOR signaling pathway^35, 36^. We therefore determined the effect of cryptolepine on mTOR signaling. Treatment of A375 and Hs294t melanoma cells with cryptolepine greatly reduced the total protein levels of mTOR and its phosphorylation in a concentration-dependent manner (Fig. 5b). Moreover, cryptolepine treatment resulted in reduced phosphorylation of the p70S6K and 4E-BP1 proteins (Fig. 5b), which are considered to be crucial downstream targets in mTOR-regulated protein synthesis. The levels of total p70S6K and 4E-BP1 protein were not significantly affected by cryptolepine. These results suggest that cryptolepine treatment reduces mTOR signaling and suggest that this reduces protein synthesis in the melanoma cells.

### Cryptolepine reduces mitochondrial biogenesis in melanoma cells

**Figure 6:**
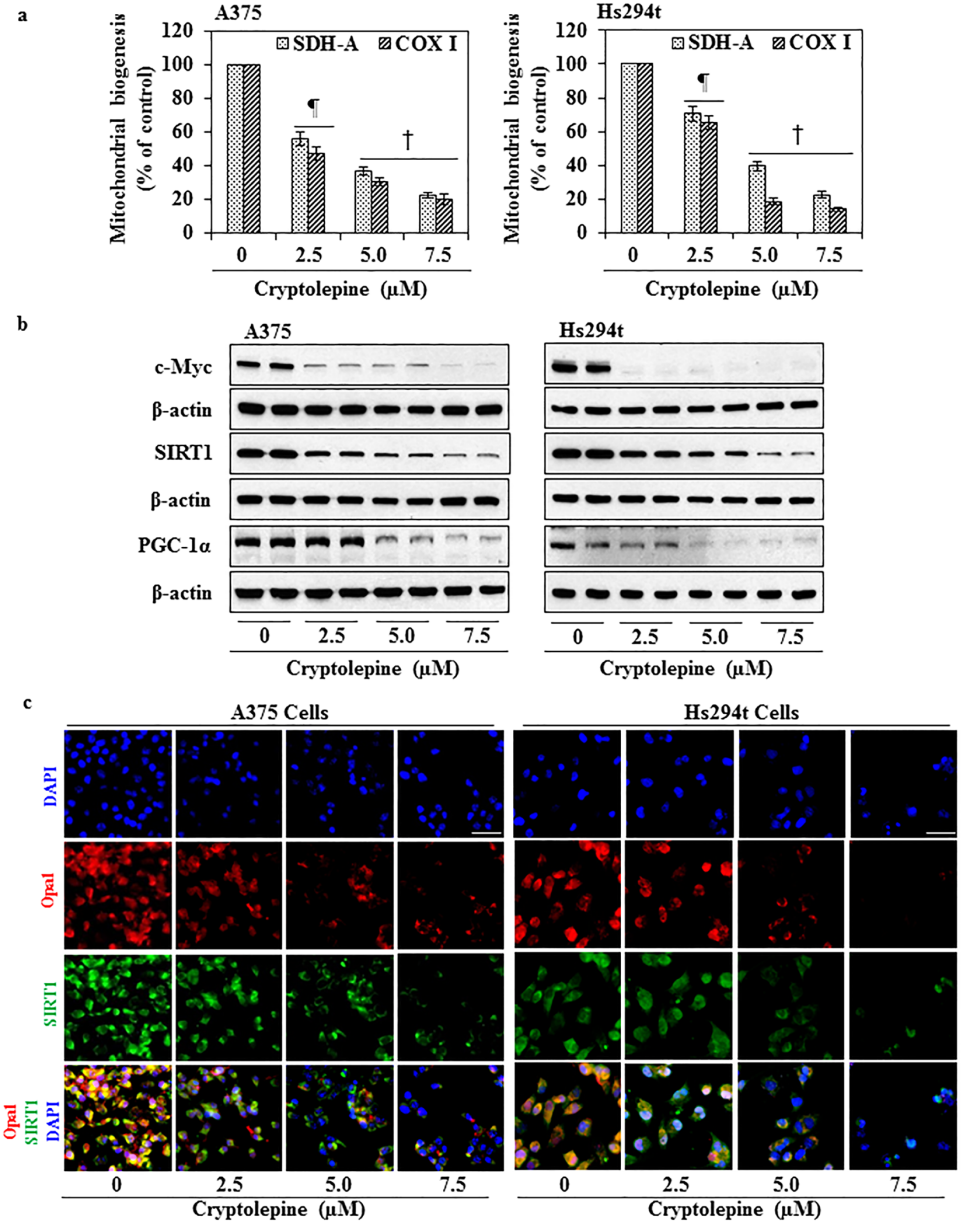
Treatment with cryptolepine inhibits mitochondrial biogenesis and associated molecular targets in melanoma cells. (**a**) Mitochondrial biogenesis in cryptolepine-treated and non-treated A375 and Hs294t cells was determined by measuring the levels of SDH-A and COX-I protein expression using the Mitochondrial Biogenesis Kit according to the manufacturer’s protocol. The experiment was performed two times. Protein expression in the vehicle-treated control group was considered as 100%. Statistical significance versus control, ^¶^ *P* < 0.01 and ^†^ *P* < 0.001. **(b)** After treatment of cells with cryptolepine for 24 h, total cell lysates from A375 and Hs294t cells were prepared and subjected to western blot analysis to determine the levels of proteins involved in mitochondrial biogenesis. Equal loading of proteins was verified by stripping the membrane and reprobing with β-actin antibody. **(c)** Immunofluorescence staining for the detection of Opa1-positive and SIRT1-positive cells was performed as detailed in Materials and Methods. Photomicrographs were obtained using Keyence Fluorescence Microscope BZ-X710 (Keyence Corporation of America). Representative photomicrographs are shown.

To evaluate the effect of cryptolepine on mitochondrial biogenesis in melanoma cells, the levels of two mitochondrial proteins succinate dehydrogenase-A (SDH-A), which is a subunit of respiratory Complex II and encoded by nuclear DNA, and cytochrome c oxidase I (COX-I), which is a subunit of respiratory Complex IV and is encoded by mitochondrial DNA, were measured simultaneously using the MitoBiogenesis™ In-Cell ELISA Colorimetric system. A375 and Hs294t cells were treated with 2.5, 5.0 and 7.5 µM cryptolepine for 24 h prior to the assay. Treatment with cryptolepine significantly reduced the levels of nuclear DNA-encoded SDH-A protein in a concentration dependent manner in A375 cells (44 to 78%; *P* < 0.001) and in Hs294t cells (29 to 78%; *P* < 0.01 to *P* < 0.001) as compared to vehicle-treated control cells (Fig. 6a). Similar effects also were observed in terms of the mitochondrial DNA-encoded COX-I protein levels in A375 cells (53 to 80%; *P* < 0.001) and Hs294t cells (35 to 86%; *P* < 0.01 to *P* < 0.001) after cryptolepine treatment as compared to vehicle-treated control cells (Fig. 6a). These results clearly demonstrated that cryptolepine significantly inhibits mitochondrial biogenesis in both of these melanoma cell lines by targeting nuclear and mitochondrial signaling pathways of mitochondrial biogenesis.

### Cryptolepine reduces protein levels of mitochondrial biogenesis regulators

PGC-1α is one of the most important central controllers of cellular bioenergetics and mitochondrial biogenesis^1, 37^. Western blot analysis revealed that treatment of A375 and Hs294t cells with cryptolepine (2.5, 5.0 and 7.5 µM) greatly reduced the levels of PGC-1α protein in a concentration-dependent manner (Fig. 6b). It has been demonstrated that SIRT1-mediated deacetylation activates PGC-1α under energy deprivation conditions resulting in enhanced mitochondrial biogenesis, cell survival and drug resistance^11, 38, 39^. We found that cryptolepine treatment reduced the levels of SIRT1 and Opa1 proteins in A375 and Hs294t cells (Fig. 6b,c). It has been shown that c-Myc stimulates expression of approximately 400 nuclear-encoded mitochondrial genes which play a central role in mitochondrial biogenesis^2, 9, 40^. Relevant to our present study, we found that cryptolepine treatment markedly reduced c-Myc protein levels in A375 and Hs294t cells in a concentration-dependent manner (Fig. 6b). These data demonstrate that cryptolepine targets proteins involved in mitochondrial biogenesis.

### Administration of cryptolepine inhibits the growth of A375 melanoma tumor xenografts in athymic nude mice

**Figure 7:**
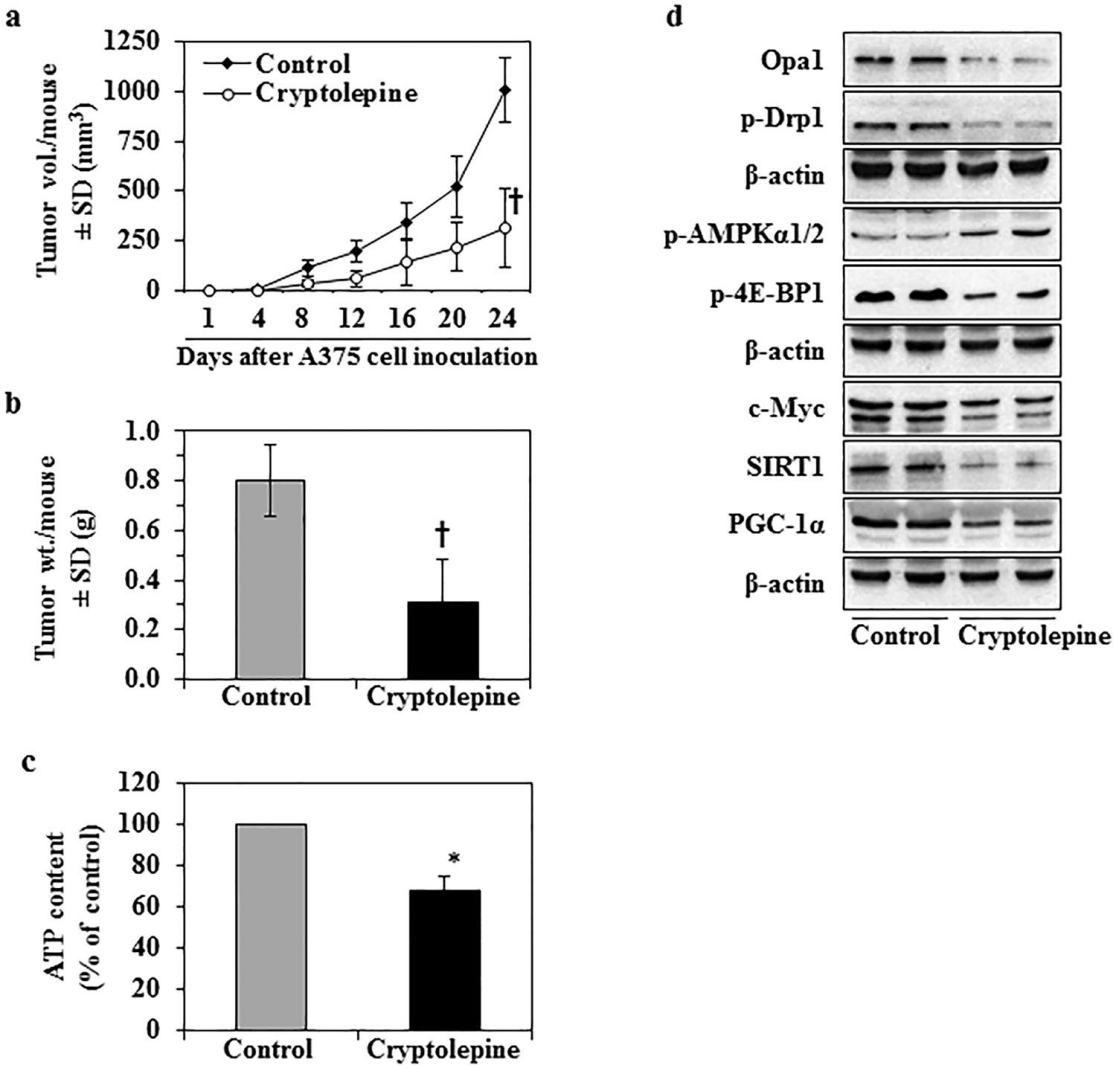
Cryptolepine treatment inhibits the growth of melanoma xenografts in athymic nude mice. The tumor xenograft growth experiment was carried out for a total of 24 d, as detailed in Materials and Methods. (**a)** Tumor xenograft growth was monitored and is shown in terms of tumor volume/mouse as a mean ± SD (mm^3^), n = 5/group. (**b**) At the termination of the experiment, tumors were harvested and the wet weight determined. (**c**) ATP content was determined in each tumor lysate sample, as described in Materials and Methods. The data are presented in terms of percentage of the non-cryptolepine-treated group and expressed as mean ± SD. *P < 0.05, ^†^ *P* < 0.001. **(d)** Levels of proteins associated with mitochondrial biogenesis/dynamics were determined in lysates of the excised A375 melanoma tumor xenografts by western blot analysis. Each sample per group was prepared by mixing the tumor tissues from 2–3 tumors from different mice. Equal loading of proteins was verified by stripping the membrane and reprobing with anti-β-actin antibodies.

To assess whether the effects of cryptolepine we observed in the *in vitro* studies are translatable to an *in vivo* system, we determined the effects of administration of cryptolepine in a melanoma xenograft model. The A375 cell line was chosen as a representative melanoma cell line as we had found similar effects of cryptolepine on the viability of the different melanoma cell lines (Fig. 1). The A375 melanoma cells were implanted in the flanks of athymic nude mice and cryptolepine was administered intraperitoneally (*i.p*.) for 24 d. The dose of cryptolepine (10 mg/Kg body weight) was based on the doses used for intraperitoneal administration of some other phytochemicals in experiments to evaluate their anti-carcinogenic effects. Monitoring of the estimated volume of the tumor xenografts indicated that the average tumor growth/size in terms of total tumor volume/mouse was significantly lower (68%, *P* < 0.001) in the cryptolepine-treated group than the group of mice administered vehicle alone (Fig. 7a). As shown in Fig. 7b, the average wet weight of the tumor at the termination of the experiment in the mice that were treated with cryptolepine was significantly lower than that of tumors from vehicle-treated control mice (61%, *P* < 0.001).

The body weight of the animals was recorded on a weekly basis for the duration of the experiment. No significant differences in the average body weight of the vehicle-treated mice and that of the cryptolepine-treated mice were observed (data not shown). In addition, cryptolepine-treated mice did not exhibit any physical sign of toxicity or abnormal behavior as compared to control mice. These data suggest that cryptolepine treatment at the concentration used in the present study is not associated with any apparent sign of toxicity in mice.

### Administration of cryptolepine disrupts mitochondrial dynamics and mitochondrial biogenesis in melanoma tumor xenografts in athymic nude mice

We further evaluated the effect of cryptolepine on various biomarkers of regulation of mitochondrial dynamics and biogenesis in the tumor xenograft tissues. The content of ATP was significantly lower (32%, *P* < 0.05) in tumor tissues of mice treated with cryptolepine compared to the tumor tissues of control mice which were treated with vehicle alone. (Fig. 7c). Cryptolepine treatment also resulted in enhanced phosphorylation of AMPKα1/2 as well as reduced phosphorylation of 4E-BP1 protein, which is a downstream effector of mTOR signaling. These data demonstrate that cryptolepine has the ability to activate the metabolic tumor suppressor and disrupt energy homeostasis signaling under *in vivo* conditions and suggest that it does so by modulating cross-talk between AMPKα1/2 and mTOR cross-talk. Western blot analysis revealed that administration of cryptolepine to A375 xenograft-bearing mice resulted in a decrease in the levels of phosphorylated form of Drp1 protein that is involved in maintenance of mitochondrial dynamics (Fig. 7d). Further, the levels of c-Myc, SIRT1 and PGC-1α protein were reduced in the tumor samples from mice treated with cryptolepine as compared with the tumor samples from vehicle-treated control mice (Fig. 7d). These results verified our *in vitro* findings and demonstrated that cryptolepine-induced effects in melanoma cells are translatable to *in vivo* conditions.

## Discussion

The balance between mitochondrial energy production and physiological functions required for cell survival is regulated by mitochondrial dynamics^41^. Maintenance of mitochondrial mass and the numbers of mitochondria in cells is regulated by the processes of mitochondrial biogenesis, fission, fusion and mitophagy. Uncontrolled mitochondrial function and dysregulated mitochondrial dynamics contribute to the pathogenesis of various diseases^42^. Thus, the targeting of mitochondrial biogenesis and mitochondrial functions has emerged as a novel preventive and therapeutic strategy for various metabolic diseases including cancer^6, 43^. Cryptolepine has been shown to possess anti-inflammatory activity and cytotoxic potential that is mediated by direct and indirect interactions with DNA^22–27, 44, 45^. In the current study, we found that cryptolepine treatment induced a highly significant decrease in melanoma cell viability and growth demonstrating that this compound possesses strong anti-melanoma activity. Furthermore, we found that cryptolepine targets mitochondrial dynamics and biogenesis in melanoma cells and that these effects were accompanied by activation of AMPKα1/2-LKB1, inhibition of mTOR signaling, and a reduction in the levels of c-Myc, SIRT1 and PGC-1α protein.

AMPKα1/2 is recognized as a central energy-sensing protein that regulates glucose and lipid metabolism and can be activated by various stress-related factors such as ATP depletion, low glucose levels, exercise and fasting^13, 46^. A growing body of evidence demonstrates that loss of AMPKα1/2 expression is associated with enhanced tumorigenesis whereas induction of AMPKα1/2 expression is related to reduced cancer cell growth^13, 14^. Activation of AMPKα1/2 has emerged as a novel strategy for prevention and treatment of cancer and several metabolic diseases^13, 14, 47^. Our data demonstrate that cryptolepine reduces ATP production in melanoma cells and enhances both the levels of AMPKα1/2 protein and its phosphorylation. We also found that expression of LKB1, an upstream regulator of AMPKα1/2^13, 48^, was enhanced in melanoma cells after cryptolepine treatment. It has been demonstrated that in response to energy-deprived conditions, activation of AMPKα1/2 inhibits protein synthesis through inhibition of mTOR signaling^35, 36^. Our results are in line with these observations in that we found that cryptolepine treatment induced activation of AMPKα1/2 in melanoma cells and caused inhibition of the protein synthesis machinery by reducing the phosphorylation of mTOR, p70S6K and 4E-BP1. Collectively, these results suggest that the ability of cryptolepine to induce activation of AMPKα1/2-LKB1 results in inhibition of mTOR signaling.

In addition to ATP depletion, we observed that cryptolepine promotes loss of mitochondrial membrane potential in melanoma cells. These effects of cryptolepine were accompanied by a greater reduction in mitochondrial content in the treated melanoma cells suggesting modulation of mitochondrial dynamics. Cells maintain the numbers of mitochondria by their continuous fission and fusion. Mitochondrial fusion is regulated by a variety of proteins, including Mfn1, Mfn2, and Opa1, whereas mitochondrial fission is associated with Drp1 and mitochondrial fission 1 protein (Fis1)^2, 3, 49^. Higher expression of Mfn1 and Mfn2 has been linked with cancer cell proliferation, enhanced cell survival and invasion. Conversely, inhibition of Mfn1 and Mfn2 expression inhibits cell growth and induces apoptosis of various cancer cells^29, 30, 43, 50^. Expression of Opa1 also has been shown to enhance cancer cell proliferation and survival whereas its inhibition leads to apoptosis^51, 52^. We found that the levels of Mfn1 and Mfn2 and Opa1 protein were considerably reduced in cryptolepine treated melanoma cells. In terms of the fission-related Drp1 protein, it has been shown that activation and enhanced expression of Drp1 enhances cancer cell proliferation, survival and drug resistance to melanoma-targeted therapies^3, 28, 53, 54^. In the present study, we found that cryptolepine treatment reduced the levels of Drp1 protein as well as its phosphorylation in melanoma cells. Collectively, these results suggest that cryptolepine-induced mitochondrial fission in concert with reduced fusion caused mitochondrial depletion in cryptolepine-treated melanoma cells.

Mitochondrial biogenesis is controlled by both nuclear genome-encoded proteins and mitochondrial genome-encoded proteins. Our study demonstrates that treatment of melanoma cells with cryptolepine resulted in reduced levels of SDH-A protein (a nuclear genome-encoded protein which is a component of Complex II enzyme) as well as COX-I protein (a mitochondrial genome-encoded protein, which is a subunit of the COX-IV enzyme complex). PGC-1α, a transcriptional coactivator, is considered to be a master regulator of mitochondrial biogenesis. PGC-1α maintains energy homeostasis and controls bioenergetics through ATP production and mitochondrial biogenesis, thereby promoting cell proliferation and survival. Enhanced expression of PGC-1α has been associated with cancer development and progression^17, 55, 56^. In our study, we found that cryptolepine greatly reduced protein expression of PGC-1α, which may have resulted in the significant depletion of ATP in cryptolepine-treated melanoma cells. SIRT1 controls mitochondrial biogenesis through deacetylation and activation of PGC-1α^11, 39, 57^ and overexpression of SIRT1 has been shown to promote melanoma cell proliferation and drug resistance^11, 58, 59^. We found that cryptolepine treatment markedly reduced the levels of SIRT1 protein in melanoma cells. Activated mTOR kinase regulates mitochondrial biogenesis both at the transcriptional level, through activation of PGC-1α/Yin Yang 1 (YY1) signaling leading to mitochondrial gene expression, and at the translational level, through repression of 4E-BPs that downregulate nuclear-encoded mitochondrial protein translation^60^. Thus, our data provide ample evidence that cryptolepine-induced inhibition of mTOR signaling and SIRT1 protein levels resulted in reduction of PGC-1α protein levels and mitochondrial biogenesis in cryptolepine-treated melanoma cells. c-Myc, a proto-oncogene that globally regulates key functions in cell growth, cell cycle, cell survival, protein synthesis, cell adhesion, cell metabolism and differentiation is recognized as the most important and central regulator of mitochondrial biogenesis^2, 9, 40^. We found that cryptolepine treatment greatly reduced the levels of c-Myc protein in melanoma cells. Importantly, the *in vitro* growth inhibitory potential and effects of cryptolepine on mitochondrial dynamics and biogenesis also were not only observed under *in vitro* conditions but also *in vivo* in a melanoma xenograft growth model in nude mice without any apparent sign of toxicity. These *in vivo* studies further verified that administration of cryptolepine inhibits tumor growth by modulating AMPKα1/2/mTOR, and reducing the c-Myc/SIRT1/PGC-1α signaling cascade involved in mitochondrial dynamics and biogenesis.

**Figure 8:**
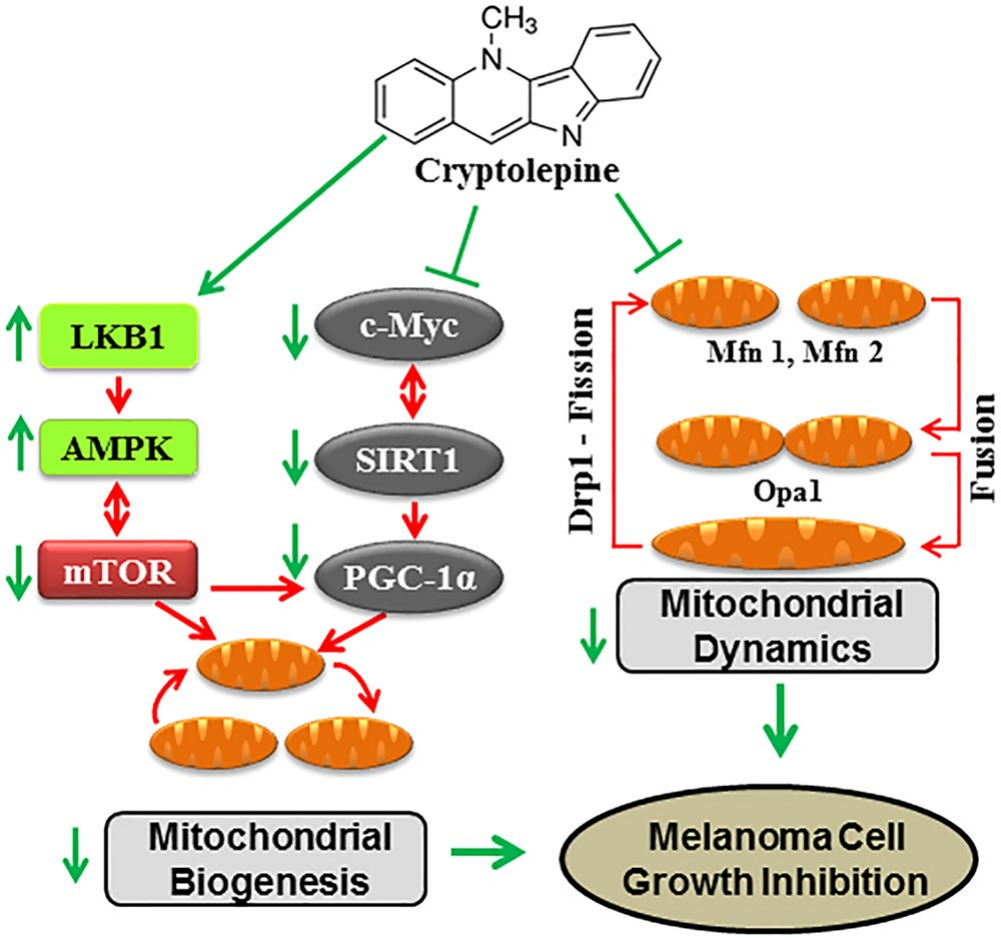
Schematic diagram showing the effects of cryptolepine on mitochondrial dynamics, biogenesis and the regulatory cascade of events resulting in suppression of melanoma cell growth. The process of melanomagenesis is influenced by dysregulated mitochondrial dynamics and mitochondrial biogenesis associated with dysregulation of regulatory molecules. The dysregulated events in melanoma cells are shown with red arrows, the therapeutic effects of cryptolepine, which result in inhibition of melanoma cell growth, are shown with green arrows.

In summary, the association of dysregulation of mitochondrial dynamics with cancer and other diseases are prompting the development of strategies to prevent or treat these diseases by targeting cellular energetics and mitochondrial biogenesis. Various phytochemicals, including flavonoids, grape polyphenol (resveratrol), green tea polyphenol (EGCG), honokiol, curcumin and berberine, that have demonstrated significant anticancer potential have been shown to target bioenergetics and activation of the metabolic tumor suppressor AMPKα1/2 and other signaling pathways involved in tumor growth and progression^14, 61, 62^. Interestingly, honokiol, a phytochemical derived from *Magnolia grandiflora*, also showed activity in vemurafenib-resistant melanoma through induction of respiratory enzyme succinate dehydrogenase in *in vivo* model^63^. In other tumor models, honokiol treatment increases AMP-activated protein kinase phosphorylation in breast cancer cells and thus inhibits the migration and invasion of breast cancer cells^64^^.^ The effect of honokiol on mitochondria was also investigated in cardiac hypertrophy. The anti-hypertrophic effects of honokiol depends on activation of the deacetylase Sirt3. Honokiol treatment increases mitochondrial rate of oxygen consumption and reduces reactive oxygen species synthesis and thus affect the cardiac hypertrophy^65^. In the present study, by employing *in vitro* cell culture and an *in vivo* melanoma xenograft model, we found that an alkaloid isolated from the roots of *Cryptolepis sanguinolenta* significantly inhibits mitochondrial biogenesis in melanoma cells by inducing mitochondrial fission, reducing enhancers of mitochondrial biogenesis and affecting mTOR signaling, as summarized in Fig. 8. The results of our study suggest more detailed pre-clinical studies as a basis for exploration of cryptolepine as a potential anti-melanoma agent.

## Materials and Methods

### Chemicals, reagents and antibodies

Purified cryptolepine hydrate (≥98% purity by HPLC), MTT dye, 2′,7′-dichlorofluorescin diacetate, and Rhodamine 123 were purchased from Sigma-Aldrich (St. Louis, MO). The MitoBiogenesis In-cell ELISA kit was purchased from Abcam (Cambridge, MA). Antibodies against c-Myc, p-Drp1, LKB1, and AMPKα1/2 were purchased from Cell Signaling Technology (Beverly MA). The Opa1 antibody was obtained from Thermo Scientific (Rockford, IL). Antibodies against PGC-1α, Mitofusin 1, Mitofusin 2, Drp1, p-AMPKα1/2, SIRT1, and β-actin and horseradish peroxidase (HRP)-labeled anti-mouse and anti-rabbit were purchased from Santa Cruz Biotechnology, Inc. (Santa Cruz, CA). The ATP Determination kit, MitoTracker Red CMXRos, AlexaFluore 488- and 594-conjugated secondary antibodies were obtained from Molecular Probes (Eugene, OR).

### Cells and cell culture conditions

The human melanoma cell lines A375 (human skin amelanotic malignant melanoma cell line) and Hs294t (human skin amelanotic melanoma cells derived from metastatic site lymph node) were obtained from the American Type Culture Collection (Manassas, VA). SK-Mel28 and SK-Mel119 were obtained from Dr. Alan Houghton of the Sloan-Kettering Institute for Cancer Research (New York, NY). Normal human epidermal melanocytes (NHEM) were obtained from Dr. Slominski of the University of Alabama at Birmingham, AL. Cells were cultured and maintained in Dulbecco’s Modified Eagle’s Medium (DMEM) or RPMI-1640 medium supplemented with 10% heat-inactivated fetal bovine serum (FBS) and 100 mg/ml penicillin-streptomycin solution at 37 ^o^C in a 95% humidified incubator with 5% CO_2_. NHEM were grown in melanocyte growth medium (MGM-4 Bullet kit; CC-3249) purchased from Lonza Walkersville Inc. (Walkersville, MD) that contains melanocyte basal cell medium (MBM-4; CC-3250) and is supplied with a supplements and growth factors kit (MGM-4 singlequots; CC-4435) including CaCl_2_, hFGF-B, PMA, rh-insulin, hydrocortisone, BPE, FBS and Gentamycin/Amphotericin-B. The cells were maintained under standard cell culture conditions as described above. All the cell lines were used 3-4 passage in each experiment. When the cells reached approximately 60–70% confluence, the media was replaced with media containing the specified concentrations of cryptolepine or vehicle control. The cryptolepine was dissolved in dimethylsulfoxide (DMSO) prior to addition to the media such that the final concentration of DMSO in the media was not more than 0.1% (v/v). An equivalent amount of DMSO was added to the culture media of control groups.

### Cell viability assay

The effects of cryptolepine on melanoma cell viability were determined using the MTT assay as described previously^66^. The viability of cryptolepine-treated cells was compared to the viability of vehicle-treated control cells, which was arbitrarily defined as 100%.

### Colony formation assay

Colony formation assay was performed as described previously^66^ to evaluate the clonogenic potential of melanoma cells after treatment with cryptolepine. Briefly, 500 cells from each of cryptolepine-treated groups (0, 1.0, 2.5, 5.0 and 7.5 µM for 24 h) were suspended in 3 ml complete growth medium and plated individually in separate wells of a 6-well plate. Cells were allowed to grow for a total 14 days with media being replaced on day 7. On day 14, colonies were washed with chilled PBS buffer, and fixed in chilled methanol for 10 min. Colonies were stained with 0.5% crystal violet (prepared in 25% methanol) for 10 min and excess stain removed by washing with water. The plates were air dried then scanned and microscopically observed.

### Analysis of mitochondrial membrane potential

The changes in mitochondrial membrane potential in cryptolepine-treated and non-treated melanoma cells were determined based on uptake of Rhodamine 123 as described previously^67^. Approximately 2 × 10^5^ cells were treated with 0, 2.5, 5.0 and 7.5 µM cryptolepine for 24 h. The cells were then incubated with Rhodamine 123 for 30 min, harvested, washed with PBS and resuspended in PBS for analysis of mitochondrial membrane potential using BD Accuri C6 flow cytometer (San Jose, CA).

### ATP content determination assay

To determine the effect of cryptolepine on ATP production by mitochondria, total ATP content was measured in total cell lysates using the ATP Determination Kit purchased from Molecular Probes following the manufacturer’s protocol. Briefly, equal number of A375 or Hs294t cells was treated with 0, 2.5, 5.0 and 7.5 µM cryptolepine for 24 h and total cell lysates prepared. An equal amount of protein (20 µg) from each treatment group was used to determine the ATP content in terms of nMol ATP/mg protein. The data are presented in terms of the percentage of the values from the vehicle-treated control cells.

### Determination of mitochondrial biogenesis

The effect of cryptolepine on mitochondrial biogenesis in melanoma cells was determined using the MitoBiogenesis In-Cell ELISA (Colorimetric) kit (MitoBiogenesis) and as described in the manufacturer’s protocol manual. Using this kit, the levels of two mitochondrial proteins, SDH-A and COX-I, were measured simultaneously in cryptolepine-treated and non-treated cells. Briefly, approximately 2 × 10^4^ cells/well were plated in each well of 96-well culture plates, 24 h later the cells were treated with different concentrations of cryptolepine (0, 2.5, 5.0 and 7.5 µM) for 24 h, and thereafter the manufacturer’s protocol was followed.

### Preparation of cell lysates and western blotting

Melanoma cells were harvested after 24 h of incubation with or without cryptolepine and cell lysates prepared as described previously^66^. Equal amounts of proteins were resolved electrophoretically on Tris-glycine gels and transferred onto a nitrocellulose membrane. Non-specific sites were blocked by incubating the membrane with blocking buffer for 1 h. The membrane was incubated with specific primary antibodies overnight at 4 °C followed by 2 h incubation with HRP-conjugated secondary antibodies. The bands were visualized by chemiluminescence on X-ray film. Equal loading of proteins was verified by probing the stripped membrane with anti-β-actin or anti-vinculin antibodies. All *in vitro* immunoblot data are presented from two independent experiments, and samples were run simultaneously on the same gel. In some cases, the membrane was cut into 2 or 3 pieces based on the molecular weights of the proteins and then blotting was performed individually. In these cases, the β-actin loading control remains common to all the proteins identified.

### Immunofluorescence staining

Approximately 5 × 10^4^ cells/well were seeded in four-well chambered slides and the next day the cells were treated with 0, 2.5, 5.0 and 7.5 µM of cryptolepine for 24 h. For mitochondrial staining, cells were further incubated with 100 nM MitoTracker Red (Molecular Probes) dye in culture medium. After incubation, the cells were washed twice with chilled PBS, fixed with 4% paraformaldehyde for 20 min and permeabilized with 0.5% Triton-X 100 in PBS for 3 min. Non-specific binding was blocked by incubating the cells with 3% BSA in PBS for 30 min. Cells were incubated with specific primary antibodies over night at 4 °C, washed three times with PBS and then incubated for 1 h with fluorochrome-conjugated secondary antibodies. After washing with PBS, the slides were mounted with Vectashield mounting media containing DAPI and analyzed and imaged using a Keyence BZ-X710 fluorescence microscope (Keyence Corporation of America, Atlanta, GA).

### Melanoma tumor xenograft model

Female athymic nude mice of 4–5 weeks of age were purchased from the National Cancer Institute (Bethesda, MD, USA). Mice were housed in the Animal Resource Facility at the University of Alabama at Birmingham in accordance with the Institutional Animal Care and Use Committee (IACUC) guidelines. All methods were performed in accordance with the guidelines and regulations of IACUC. The tumor xenograft protocol was approved by the IACUC of the University of Alabama at Birmingham. To determine the *in vivo* chemotherapeutic efficacy of cryptolepine against human melanoma xenograft growth, exponentially growing A375 melanoma cells (2 × 10^6^ A375 cells in 50 μl PBS + 50 μl matrigel) were injected subcutaneously in each flank of each mouse. One day after tumor cell inoculation, animals were divided randomly into two groups with five mice per group. Group 1 mice (vehicle-treated control group) were administered 100 μl of vehicle (DMSO: water; 1:1 volume/volume) *i.p*. Group II mice were administered cryptolepine (10 mg/kg body weight) *i.p*. in the same volume of vehicle solution as the control group. All treatments were given 3 days/week (Mon, Wed and Fri) and the experiment was terminated at d 24 after tumor cell inoculation. The tumor growth and body weight per mouse in each group were recorded on every fourth day. Tumor size was measured using Vernier calipers and volumes were calculated using the hemiellipsoid model formula: tumor volume = 1/2 (4π/3) (l/2) (w/2) h, where l = length, w = width and h = height. At the termination of the experiment, mice were sacrificed, the tumor from each mouse was excised and the wet weight of each tumor in each group was recorded. Tumors were pooled to prepare tumor lysates for western blot analysis.

### Statistical analysis

The statistical significance of the difference between the values of control and treatment groups was determined by a student-t test and using GraphPad Software, University of California, San Diego, CA. In each case, *P* < 0.05 was considered as statistically significant.
